# Noncommutative Bohnenblust–Hille inequalities

**DOI:** 10.1007/s00208-023-02680-0

**Published:** 2023-07-24

**Authors:** Alexander Volberg, Haonan Zhang

**Affiliations:** 1 Department of Mathematics, MSU, East Lansing, MI 48823 USA; 2 Hausdorff Center of Mathematics, Bonn, Germany; 3 Department of Mathematics, University of California, Irvine, CA 92617 USA; 4 Institute of Science and Technology Austria (ISTA), Am Campus 1, 3400 Klosterneuburg, Austria

**Keywords:** 47A30, 81P45, 06E30

## Abstract

Bohnenblust–Hille inequalities for Boolean cubes have been proven with dimension-free constants that grow subexponentially in the degree (Defant et al. in Math Ann 374(1):653–680, 2019). Such inequalities have found great applications in learning low-degree Boolean functions (Eskenazis and Ivanisvili in Proceedings of the 54th annual ACM SIGACT symposium on theory of computing, pp 203–207, 2022). Motivated by learning quantum observables, a qubit analogue of Bohnenblust–Hille inequality for Boolean cubes was recently conjectured in Rouzé et al. (Quantum Talagrand, KKL and Friedgut’s theorems and the learnability of quantum Boolean functions, 2022. arXiv preprint arXiv:2209.07279). The conjecture was resolved in Huang et al. (Learning to predict arbitrary quantum processes, 2022. arXiv preprint arXiv:2210.14894). In this paper, we give a new proof of these Bohnenblust–Hille inequalities for qubit system with constants that are dimension-free and of exponential growth in the degree. As a consequence, we obtain a junta theorem for low-degree polynomials. Using similar ideas, we also study learning problems of low degree quantum observables and Bohr’s radius phenomenon on quantum Boolean cubes.

## Introduction

In 1930, Littlewood [19] proved that for any $n\ge 1$ and any bilinear form $B:{\mathbb {C}}^n\otimes {\mathbb {C}}^n\rightarrow {\mathbb {C}}$, we have

1.1 $$\begin{aligned} \left( \sum _{i,j}|B(e_i,e_j)|^{4/3}\right) ^{3/4}\le \sqrt{2}\Vert B\Vert , \end{aligned}$$

where $\{e_j,1\le j\le n\}$ is the canonical basis of ${\mathbb {C}}^n$ and $\Vert B\Vert$ denotes the norm of the bilinear form, i.e.

$$\begin{aligned} \Vert B\Vert \,{:}{=}\,\sup \{|B(x,y)|:x,y\in {\mathbb {C}}^n, \Vert x\Vert _\infty \le 1, \Vert y\Vert _\infty \le 1\}. \end{aligned}$$

Here 4/3 is optimal and (1.1) is known as *Littlewood’s 4/3 inequality*. Right after Littlewood’s proof of (1.1), Bohnenblust and Hille [1] extended this result to multilinear forms: For any $d\ge 1$, there exists a constant $C_d>0$ depending only on *d* such that for any $n\ge 1$ and any *d*-linear form $B:{\mathbb {C}}^n\times \cdots \times {\mathbb {C}}^n\rightarrow {\mathbb {C}}$ we have

1.2 $$\begin{aligned} \left( \sum _{i_1,\ldots , i_d=1}^{n}|B(e_{i_1},\ldots , e_{i_d})|^{\frac{2d}{d+1}}\right) ^{\frac{d+1}{2d}} \le C_d\Vert B\Vert , \end{aligned}$$

where $\Vert B\Vert$ is defined in a similar way as above and the exponent $2d/(d+1)$ is optimal. The inequalities (1.2) have played a key role in Bohnenblust and Hille’s solution [1] to *Bohr’s strip problem* [4] concerning the convergence of Dirichlet series. Such multilinear form inequalities (1.2) and their polynomial variants (which we shall recall for Boolean cubes) are known as *Bohnenblust–Hille inequalities*.

Since then, Bohnenblust–Hille inequalities have been extended to different contexts. Recent years have seen great progress in improving the constants in Bohnenblust–Hille inequalities (e.g. $C_d$ in (1.2)) and this has led to the resolution of a number of open problems in harmonic analysis. See for example [7, 9, 10, 13] and references therein.

In [3] Blei extended Bohnenblust–Hille inequalities to polynomials on Boolean cubes with dimension-free constants. Recently, this result was revisited by Defant et al. [12]. Moreover, they proved that the dimension-free Bohnenblust–Hille constants for Boolean cubes actually grow at most subexponentially in the degree. To state their results, recall that any function $f:\{-1,1\}^n\rightarrow {\mathbb {C}}$ has the *Fourier–Walsh expansion*:

$$\begin{aligned} f(x)=\sum _{S\subset [n]}\widehat{f}(S)\chi _S(x), \end{aligned}$$

where for each $S\subset [n]\,{:}{=}\,\{1,\ldots ,n\}$, $\widehat{f}(S)\in {\mathbb {C}}$ and

$$\begin{aligned} \chi _S(x)\,{:}{=}\,\prod _{j\in S}x_j,\qquad x=(x_1,\ldots , x_n). \end{aligned}$$

The function *f* is said to be *of degree at most d* if $\widehat{f}(S)=0$ whenever $|S|>d$; and is said to be *d*-*homogeneous* if $\widehat{f}(S)=0$ whenever $|S|\ne d$. Defant, Mastyło and Pérez proved the following theorem (they considered real-valued functions but the proof works for complex-valued case as well).

### Theorem 1.1

[12, Theorem 1] For any $d\ge 1$, there exists $C_d>0$ such that for any $n\ge 1$ and any $f:\{-1,1\}^n\rightarrow {\mathbb {C}}$ of degree at most *d*, we have

1.3 $$\begin{aligned} \left( \sum _{|S|\le d}|\widehat{f}(S)|^{\frac{2d}{d+1}}\right) ^{\frac{d+1}{2d}}\le C_d \Vert f\Vert _\infty . \end{aligned}$$

Denoting $\text {BH}^{\le d}_{\{\pm 1\}}$ the best constant $C_d$ such that (1.3) holds, then there exists $C>0$ such that $\text {BH}^{\le d}_{\{\pm 1\}}\le C^{\sqrt{d\log d}}$. So the Bohnenblust–Hille constant $\text {BH}^{\le d}_{\{\pm 1\}}$ is of subexponential growth.

As we mentioned, Blei [3] first proved $\text {BH}^{\le d}_{\{\pm 1\}}<\infty$. One of main contributions of Defant et al. [12] lies in this subexponential bound $\text {BH}^{\le d}_{\{\pm 1\}}\le C^{\sqrt{d\log d}}$. Recently, this Bohnenblust–Hille inequality for Boolean cubes (1.3) has found great applications in learning bounded low-degree functions on Boolean cubes [14], which will be explained in more detail in Sect. 4. In view of this, an analogue of (1.3) for qubit systems was conjectured in [20], motivated by learning quantum observables following the work of Eskenazis and Ivanisvili [14]. But actually this quantum analogue of Bohnenblust–Hille inequality was already contained in a result of Huang, Chen and Preskill in the preprint [16] that was not online available when the conjecture was made. Their motivation is to predict any quantum process. In this paper, we provide another proof that is simpler and gives better constants. Moreover, our method is more general which allows us to reduce many problems on the qubit systems to classical Boolean cubes. We refer to Sect. 2 for more discussions and to Sect. 6 for the comparison on our results and the work in [16].

In our quantum setup, the Boolean cubes $\{-1,1\}^n$ are replaced by $M_2({\mathbb {C}})^{\otimes n}$, the *n*-fold tensor product of 2-by-2 complex matrix algebras. Recall that Pauli matrices and the identity matrix

$$\begin{aligned} \sigma _0=\begin{pmatrix}1&{}\quad 0\\ 0&{}\quad 1\end{pmatrix},\quad \sigma _1=\begin{pmatrix}0&{}\quad 1\\ 1&{}\quad 0\end{pmatrix},\quad \sigma _2=\begin{pmatrix}0&{}\quad -i\\ i&{}\quad 0\end{pmatrix},\quad \sigma _3=\begin{pmatrix}1&{}\quad 0\\ 0&{}\quad -1\end{pmatrix}, \end{aligned}$$

form a basis of $M_2({\mathbb {C}})$. For ${\textbf {s}}=(s_1,\ldots , s_n)\in \{0,1,2,3\}^n$, we put

$$\begin{aligned} \sigma _{\textbf {s}}\,{:}{=}\,\sigma _{s_1}\otimes \cdots \otimes \sigma _{s_n}. \end{aligned}$$

All the $\sigma _{{\textbf {s}}}, {\textbf {s}}\in \{0,1,2,3\}^n$ form a basis of $M_2({\mathbb {C}})^{\otimes n}$ and play the role of characters $\chi _S,S\in [n]$ in the classical case. So any $A\in M_2({\mathbb {C}})^{\otimes n}$ has the unique Fourier expansion

$$\begin{aligned} A=\sum _{{\textbf {s}}\in \{0,1,2,3\}^n} \widehat{A}_{\textbf {s}}\,\sigma _{\textbf {s}}\end{aligned}$$

with $\widehat{A}_{\textbf {s}}\in {\mathbb {C}}$ being the Fourier coefficient. For any ${\textbf {s}}=(s_1,\ldots , s_n)\in \{0,1,2,3\}^n$, we denote by $|{\textbf {s}}|$ the number of non-zero $s_j$’s. Similar to the classical setting, $A\in M_2({\mathbb {C}})^{\otimes n}$ is *of degree at most d* if $\widehat{A}_{\textbf {s}}=0$ whenever $|{\textbf {s}}|>d$, and it is *d*-*homogeneous* if $\widehat{A}_{\textbf {s}}=0$ whenever $|{\textbf {s}}|\ne d$.

In the sequel, we always use $\Vert A\Vert$ to denote the operator norm of *A*. Our main result is the following:

### Theorem 1.2

For any $d\ge 1$, there exists $C_d>0$ such that for all $n\ge 1$ and all $A=\sum _{|{\textbf {s}}|\le d}\widehat{A}_{{\textbf {s}}}\sigma _{{\textbf {s}}}\in M_2({\mathbb {C}})^{\otimes n}$ of degree at most *d*, we have

1.4 $$\begin{aligned} \left( \sum _{|{\textbf {s}}|\le d}|\widehat{A}_{{\textbf {s}}}|^{\frac{2d}{d+1}}\right) ^{\frac{d+1}{2d}} \le C_d \Vert A\Vert . \end{aligned}$$

Moreover, denote $\text {BH}^{\le d}_{M_2({\mathbb {C}})}$ the best constant $C_d>0$. Then we have $\text {BH}^{\le d}_{M_2({\mathbb {C}})}\le 3^d \text {BH}^{\le d}_{\{\pm 1\}}$, so that it is at most of exponential growth.

A special choice of polynomials yields noncommutative analogues of Bohnenblust–Hille inequalities for multilinear forms. For this we use the following notation. Fix $n\ge 1$. For $\kappa \in \{1,2,3\}$ and $i\in [n]$, we write $\sigma ^{(\kappa )}_i$ for $\sigma _{{\textbf {s}}}$ where ${\textbf {s}}=(s_1,\ldots , s_n)\in \{0,1,2,3\}^n$ with $s_i=\kappa$ and $s_j=0$ whenever $j\ne i$.

### Corollary 1.3

Fix $d\ge 1$. Then there exists $C_d>0$ such that for any $n\ge 1$ and any (each $\sigma ^{(\kappa _j)}_{i_j}\in M_2({\mathbb {C}})^{\otimes n}$)

$$\begin{aligned} A\,{:}{=}\,\sum _{\kappa _1,\ldots , \kappa _d\in \{1,2,3\}}\sum _{i_1,\ldots , i_d=1}^{n}a^{\kappa _1,\ldots , \kappa _d}_{i_1,\ldots , i_d}\sigma ^{(\kappa _1)}_{i_1}\otimes \cdots \otimes \sigma ^{(\kappa _d)}_{i_d}, \end{aligned}$$

we have

$$\begin{aligned} \left( \sum _{\kappa _1,\ldots , \kappa _d\in \{1,2,3\}}\sum _{i_1,\ldots , i_d=1}^{n}|a^{\kappa _1,\ldots , \kappa _d}_{i_1,\ldots , i_d}|^{\frac{2d}{d+1}}\right) ^{\frac{d+1}{2d}} \le C_d \Vert A\Vert . \end{aligned}$$

Moreover, $C_d\le 3^d \text {BH}^{\le d}_{\{\pm 1\}}$, and it becomes a noncommutative analogue of Littlewood’s 4/3 inequality when $d=2$.

### Remark 1.4

Note that the algebra of function on $\{-1,1\}^n$ can be viewed as a commutative subalgebra of $M_2({\mathbb {C}})^{\otimes n}$ spanned by $\sigma _{{\textbf {s}}},{\textbf {s}}\in \{0,3\}^n$. So (1.3) is a special case of (1.4)

and we always have $\text {BH}^{\le d}_{\{\pm 1\}}\le \text {BH}^{\le d}_{M_2({\mathbb {C}})}$. Our main result Theorem 1.2 states that the converse holds up to a factor $3^d$.

### Remark 1.5

The main theorem of Huang et al. [16, Theorem 5] is actually more general

which admits (1.4) as a corollary [16, Corollary 3]. Their proof is different from ours and the constant they obtained is $C_d\sim d^{\mathcal {O}(d)}$ which is worse.

Recall that a function $f:\{-1,1\}^n\rightarrow {\mathbb {C}}$ is called a *k*-*junta* if it depends on at most *k* coordinates. Similarly, a matrix $A\in M_{2}({\mathbb {C}})^{\otimes n}$ is a *k*-*junta* if it acts non-trivially on at most *k* qubits, that is,

$$ \begin{aligned} |\{1\le j\le n: \exists {\textbf {s}}\in \{0,1,2,3\}^n \quad \text {s.t.}\quad \widehat{A}_{{\textbf {s}}}\ne 0 \quad  \&  \quad s_j\ne 0\}|\le k. \end{aligned}$$

It is known that [6, 8] if a bounded function *f* over $\{-1,1\}^n$ is of low degree, then it is close to a junta. In the next corollary we derive such a result in a quantum setting. We refer to [20, Theorem 3.9] to another quantum junta type theorem related to the influences instead of the degree.

### Corollary 1.6

Suppose that $A\in M_2({\mathbb {C}})^{\otimes n}$ is of degree at most *d* and $\Vert A\Vert \le 1$. Then for any $\epsilon >0$, there exists a *k*-junta $B\in M_2({\mathbb {C}})^{\otimes n}$ such that

$$\begin{aligned} \Vert A-B\Vert _2\le \epsilon \qquad \textrm{with }\qquad k\le \frac{d\left( \text {BH}^{\le d}_{M_2({\mathbb {C}})}\right) ^{2d}}{\epsilon ^{2d}}. \end{aligned}$$

Here $\Vert \cdot \Vert _2$ denoted the Hilbert–Schmidt norm with respect to the normalized trace, that is, $\Vert A\Vert ^2_2=2^{-n}\text {tr}[A^*A]$. In particular, we may choose $k\le dC^{2d^2}\epsilon ^{-2d}$ for some universal $C>0$.

### Remark 1.7

The results in [6, 8] are more general. However, in the case when polynomials are of low degree, the proof presented here that uses Bohnenblust–Hille inequalities is simpler. We are grateful to Alexandros Eskenazis for pointing out to us this proof.

To prove Theorem 1.2, we reduce the problem to the (commutative) Boolean cube case, at a price of an extra factor $3^d$. In fact, our main contribution is a general method that reduces many problems in the quantum setting to the classical setting. This method will be explained in Sect. 2, while the proof of Theorem 1.2 and Corollary 1.6 will be presented in Sect. 3.

We shall illustrate the strength of this reduction method with two more applications. The first one concerns learning bounded low-degree quantum observables which will be Sect. 4 and the main result is Theorem 4.1. The second one is Theorem 5.2 on Bohr’s radius phenomenon in the context of quantum Boolean cubes which will be discussed in Sect. 5.

In Sect. 6, we briefly compare our results with the work of Huang et al. [16].

**Notation**. We shall use $\text {tr}$ to denote the usual (unnormalized) trace on matrix algebras, and $\langle \cdot , \cdot \rangle$ the inner product on ${\mathbb {C}}^n$ that is linear in the second argument. By $\Vert A\Vert _p$ of a *k*-by-*k* matrix *A* we always mean the normalized Schatten-*p* norm, i.e. $\Vert A\Vert _p^p=2^{-k}\text {tr}|A|^p$. For any unit vector $\eta \in {\mathbb {C}}^n$, we use $|\eta \rangle \langle \eta |$ to denote the associated rank one projection operator. Sometimes people use the convention $\eta \otimes \eta$ instead. By a density matrix we mean a positive semi-definite matrix with unit trace.

## Reduction to the commutative case

In this section, we present a general reduction method. For this let us collect a few facts about Pauli matrices. For each $j=1,2,3$, $\sigma _j$ is self-adjoint unitary, and has 1 and $-1$ as eigenvalues. We denote by $e^j_1$ and $e^j_{-1}$ the corresponding unit eigenvectors, respectively. Pauli matrices $\sigma _j, j=1,2,3$ satisfy the following anticommutation relation:

2.1 $$\begin{aligned} \sigma _j\sigma _k+\sigma _k\sigma _j=0, \qquad j\ne k\in \{1,2,3\}. \end{aligned}$$

We record the following simple fact as a lemma.

### Lemma 2.1

For any $j, k\in \{1,2,3\}$ and $\epsilon \in \{-1,1\}$, we have

2.2 $$\begin{aligned} \langle \sigma _j e^{k}_{\epsilon },e^{k}_{\epsilon }\rangle =\delta _{jk}\epsilon . \end{aligned}$$

### Proof

When $j=k$, (2.2) is trivial by definition of $e^k_\epsilon$. When $j\ne k$, by (2.1) we have

$$\begin{aligned} \epsilon \langle \sigma _j e^{k}_{\epsilon },e^{k}_{\epsilon }\rangle =\langle \sigma _j \sigma _k e^{k}_{\epsilon },e^{k}_{\epsilon }\rangle =-\langle \sigma _k \sigma _j e^{k}_{\epsilon },e^{k}_{\epsilon }\rangle =-\epsilon \langle \sigma _j e^{k}_{\epsilon },e^{k}_{\epsilon }\rangle . \end{aligned}$$

This gives (2.2) for $j\ne k$ since $\epsilon \ne 0$. $\square$

Recall that $A\in M_2({\mathbb {C}})^{\otimes n}$ has the form

2.3 $$\begin{aligned} A=\sum _{{\textbf {s}}\in \{0,1,2,3\}^n}\widehat{A}_{{\textbf {s}}}\sigma _{{\textbf {s}}}. \end{aligned}$$

It will be more convenient for us to rewrite it as

2.4 $$\begin{aligned} A=\sum _{l\ge 0}\sum _{\kappa _1,\ldots , \kappa _l\in \{1,2,3\}}\sum _{1\le i_1<\cdots <i_l\le n}a^{\kappa _1,\ldots , \kappa _l}_{i_1,\ldots , i_l}\sigma ^{\kappa _1,\ldots , \kappa _l}_{i_1,\ldots , i_l} \end{aligned}$$

where to each $(l;\kappa _1,\ldots , \kappa _l;i_1<\cdots <i_l)$, we associate it with ${\textbf {s}}=(s_1,\ldots , s_n)\in \{0,1,2,3\}^n$ of length $|{\textbf {s}}|=l$ with

$$\begin{aligned} s_{k}= {\left\{ \begin{array}{ll} \kappa _j,&{} k=i_j, 1\le j\le l\\ 0,&{} \text {otherwise} \end{array}\right. } \end{aligned}$$

so that

$$\begin{aligned} \widehat{A}_{{\textbf {s}}}=a^{\kappa _1,\ldots , \kappa _l}_{i_1,\ldots , i_l}\qquad \text {and} \qquad \sigma _{{\textbf {s}}}=\sigma ^{\kappa _1,\ldots , \kappa _l}_{i_1,\ldots , i_l}. \end{aligned}$$

In other words, $\sigma ^{\kappa _1,\ldots , \kappa _l}_{i_1,\ldots , i_l}$ is defined as

$$\begin{aligned} \sigma ^{\kappa _1,\ldots , \kappa _l}_{i_1,\ldots , i_l}\,{:}{=}\,\cdots \otimes \sigma _{\kappa _1}\otimes \cdots \otimes \sigma _{\kappa _l}\otimes \cdots , \end{aligned}$$

where $\sigma _{\kappa _j}$ appears in the $i_j$-th place for each $1\le j\le l$, and all other $(n-l)$ components are simply identity matrices $\sigma _0$.

The main ingredient of our method is the following Proposition 2.2. To make the statement brief, we shall use the map

$$\begin{aligned} q=q_n:\{{\textbf {s}}\in \{0,1,2,3\}^n\}\rightarrow \{S\subset [3n]\}, \end{aligned}$$

that realizes the above identification of (2.3) and (2.4). If ${\textbf {s}}=\textbf{0}$ is the 0 vector, then we define $q(\textbf{0})\,{:}{=}\,\emptyset$. Each ${\textbf {s}}=(s_1,\ldots , s_n)\in \{0,1,2,3\}^n$ with $1\le |{\textbf {s}}|=l\le n$ is assigned to a tuple $(\kappa _1,\ldots , \kappa _l;i_1<\cdots < i_l)\in [3]^l\times [n]^l$ with $s_{i_j}=\kappa _j$. Then we define

$$\begin{aligned} q({\textbf {s}})\,{:}{=}\,\{n(\kappa _j-1)+i_j:1\le j\le l\}=:S. \end{aligned}$$

For example, if $n=5$ and ${\textbf {s}}=(0,1,2,3,1)$, then

$$\begin{aligned} q({\textbf {s}})=S=\{2, 5+3, 10+4, 5\}\subset [15]. \end{aligned}$$

Note that $|S|=|{\textbf {s}}|=l$. So

$$\begin{aligned} q\left( \{{\textbf {s}}\in \{0,1,2,3\}^n:|{\textbf {s}}|\le d\}\right) \subset \{S\subset [3n]:|S|\le d\}. \end{aligned}$$

Moreover, this map $q:\{0,1,2,3\}^n\rightarrow \{S\subset [3n]\}$ is injective but not surjective. We denote by $p=p_n$ its inverse over $\text {Im}(q)\subset 2^{[3n]}$ (here and in what follows, we use $2^{[3n]}$ to denote the family of subsets of [3*n*])

$$\begin{aligned} {\textbf {s}}=p(S), \quad S\in \text {Im}(q)\subset 2^{[3n]}. \end{aligned}$$

Therefore, the above formulae (2.3) and (2.4) can also be rewritten as

2.5 $$\begin{aligned} A=\sum _{S\in \text {Im}(q)\subset 2^{[3n]}} \widehat{A}_{p(S)}\sigma _{p(S)}. \end{aligned}$$

### Proposition 2.2

Fix $n\ge 1$. There exists a family

$$\begin{aligned} \mathcal {S}=\mathcal {S}_n=\{\rho ({\epsilon }):{\epsilon }\in \{-1,1\}^{3n}\} \end{aligned}$$

of density matrices in $M_2({\mathbb {C}})^{\otimes n}$ such that the following holds: For any $A\in M_2({\mathbb {C}})^{\otimes n }$, the function $f_A:\{-1,1\}^{3n}\rightarrow {\mathbb {C}}$ defined by

$$\begin{aligned} f_A({\epsilon })=\text {tr}[A\rho ({\epsilon })],\qquad {\epsilon }\in \{-1,1\}^{3n} \end{aligned}$$

satisfies

2.6 $$\begin{aligned} \widehat{f}_A(S)= {\left\{ \begin{array}{ll} 3^{-|S|}\widehat{A}_{p(S)}, &{} S\in \text {Im}(q)\subset 2^{[3n]}\\ 0,&{} S\in 2^{[3n]}\setminus \text {Im}(q) \end{array}\right. }. \end{aligned}$$

In particular, $\Vert f_A\Vert _\infty \le \Vert A\Vert$, $\deg (f_A)=\deg (A)$ and for all $d\ge 0$

2.7 $$\begin{aligned} \left( \sum _{{\textbf {s}}\in \{0,1,2,3\}^n:|{\textbf {s}}|\le d}|\widehat{A}_{{\textbf {s}}}|^{r}\right) ^{1/r}\le 3^d\left( \sum _{S\subset [3n]:|S|\le d} |\widehat{f}_A(S)|^{r}\right) ^{1/r}, 0< r<\infty .\qquad \end{aligned}$$

### Proof

For any

$$\begin{aligned} {\epsilon }\,{:}{=}\,\left( \epsilon ^{(1)}_1,\ldots , \epsilon ^{(1)}_n,\epsilon ^{(2)}_1,\ldots , \epsilon ^{(2)}_n,\epsilon ^{(3)}_1,\ldots , \epsilon ^{(3)}_n\right) \in \{-1,1\}^{3n}, \end{aligned}$$

the matrix $\rho ({\epsilon })$ is defined as follows

$$\begin{aligned} \rho \,{:}{=}\,\rho ({\epsilon })=\rho _1\otimes \cdots \otimes \rho _n \in M_2({\mathbb {C}})^{\otimes n}, \end{aligned}$$

where for each $1\le j\le n$

$$\begin{aligned} \rho _j\,{:}{=}\,\rho _j({\epsilon })=\frac{1}{3}|e^1_{\epsilon ^{(1)}_j}\rangle \langle e^1_{\epsilon ^{(1)}_j}|+\frac{1}{3}|e^{2}_{\epsilon ^{(2)}_j}\rangle \langle e^2_{\epsilon ^{(2)}_j}|+\frac{1}{3}|e^3_{\epsilon ^{(3)}_j}\rangle \langle e^3_{\epsilon ^{(3)}_j}|. \end{aligned}$$

Recall for any $\kappa \in \{1,2,3\}$ and $\epsilon \in \{-1,1\}$, $e^\kappa _{\epsilon }$ is a unit vector. Hence each $\rho _j({\epsilon })$ is positive semi-definite with trace 1. So $\rho ({\epsilon })$ is also a density matrix.

To prove (2.6), let us employ the notation (2.4). Then for $\rho =\rho ({\epsilon })$ defined as above

$$\begin{aligned} \text {tr}[A\rho ]=\sum _{l\ge 0}\sum _{\kappa _1,\ldots , \kappa _l\in \{1,2,3\}}\sum _{1\le i_1<\cdots <i_l\le n}a^{\kappa _1,\ldots , \kappa _l}_{i_1,\ldots , i_l} \text {tr}\left[ \sigma ^{\kappa _1,\ldots , \kappa _l}_{i_1,\ldots , i_l}\rho \right] . \end{aligned}$$

By definition,

$$\begin{aligned} \text {tr}\left[ \sigma ^{\kappa _1,\ldots , \kappa _l}_{i_1,\ldots , i_l}\rho \right]&=\text {tr}[\sigma _{\kappa _1}\rho _{i_1}]\cdots \text {tr}[\sigma _{\kappa _l}\rho _{i_l}]\prod _{j\notin \{ i_1,\ldots , i_l\}}\text {tr}[\sigma _0\rho _j]\\&=\text {tr}[\sigma _{\kappa _1}\rho _{i_1}]\cdots \text {tr}[\sigma _{\kappa _l}\rho _{i_l}]. \end{aligned}$$

By Lemma 2.1, for each $1\le \kappa \le 3$ and $\epsilon \in \{-1,1\}$

$$\begin{aligned} \text {tr}\left[ \sigma _{\kappa _j}|e^{\kappa }_{\epsilon }\rangle \langle e^{\kappa }_{\epsilon }|\right] =\langle \sigma _{\kappa _j}e^{\kappa }_{\epsilon },e^{\kappa }_{\epsilon }\rangle =\epsilon \delta _{\kappa _j \kappa }. \end{aligned}$$

Thus (recall that $\kappa _j\in \{1,2,3\}$)

$$\begin{aligned} \text {tr}[\sigma _{\kappa _j}\rho _{i_j}] =\frac{1}{3}\sum _{\kappa =1}^{3}\text {tr}\left[ \sigma _{\kappa _j}|e^{\kappa }_{\epsilon ^{(\kappa )}_{i_j}}\rangle \langle e^{\kappa }_{\epsilon ^{(\kappa )}_{i_j}}|\right] =\frac{1}{3}\sum _{\kappa =1}^{3}\epsilon ^{(\kappa )}_{i_j}\delta _{\kappa _j \kappa } =\frac{1}{3}\epsilon ^{(\kappa _j)}_{i_j}. \end{aligned}$$

So we have shown that

$$\begin{aligned} \text {tr}\left[ \sigma ^{\kappa _1,\ldots , \kappa _l}_{i_1,\ldots , i_l}\rho \right] =\text {tr}[\sigma _{\kappa _1}\rho _{i_1}]\cdots \text {tr}[\sigma _{\kappa _l}\rho _{i_l}] =\frac{1}{3^l}\epsilon ^{(\kappa _1)}_{i_1}\cdots \epsilon ^{(\kappa _l)}_{i_l}, \end{aligned}$$

and thus

$$\begin{aligned} f_A({\epsilon }) =\sum _{l\ge 0}\sum _{\kappa _1,\ldots , \kappa _l\in \{1,2,3\}}\sum _{1\le i_1<\cdots <i_l\le n}\frac{1}{3^l}a^{\kappa _1,\ldots , \kappa _l}_{i_1,\ldots , i_l} \epsilon ^{(\kappa _1)}_{i_1}\cdots \epsilon ^{(\kappa _l)}_{i_l}. \end{aligned}$$

This is nothing but (2.6). To see the rest of statements, note first that by definition and Hölder’s inequality:

$$\begin{aligned} |f_A({\epsilon })|\le \text {tr}|\rho ({\epsilon })|\cdot \Vert A\Vert =\Vert A\Vert . \end{aligned}$$

The desired $\deg (f_A)=\deg (A)$ and the inequality (2.7) follow immediately from (2.6) and the fact that $|p(S)|=|S|$. $\square$

## Proof of Theorem 1.2 and Corollary 1.6

In this section we prove Theorem 1.2 and Corollary 1.6.

### Proof of Theorem 1.2

Using the notation (2.5) we need to prove

$$\begin{aligned} \left( \sum _{S\in \text {Im}(q)\subset 2^{[3n]}:|S|\le d}|\widehat{A}_{p(S)}|^{\frac{2d}{d+1}}\right) ^{\frac{d+1}{2d}}\le 3^d \text {BH}^{\le d}_{\pm 1}\Vert A\Vert \end{aligned}$$

for all

$$\begin{aligned} A=\sum _{S\in \text {Im}(q)\subset 2^{[3n]}:|S|\le d} \widehat{A}_{p(S)}\sigma _{p(S)}\in M_2({\mathbb {C}})^{\otimes n}. \end{aligned}$$

Let $f_A:\{-1,1\}^{3n}\rightarrow {\mathbb {C}}$ be the function associated to $A\in M_2({\mathbb {C}})^{\otimes n}$ constructed in Proposition 2.2. So it is also of degree at most *d*. Then the desired result follows from the following chain of inequalities:

$$\begin{aligned} \left( \sum _{S\in \text {Im}(q)\subset 2^{[3n]}:|S|\le d}|\widehat{A}_{p(S)}|^{\frac{2d}{d+1}}\right) ^{\frac{d+1}{2d}}&\le 3^d\left( \sum _{S\subset [3n]:|S|\le d} |\widehat{f}_A(S)|^{\frac{2d}{d+1}}\right) ^{\frac{d+1}{2d}}\\&\le 3^d\text {BH}^{\le d}_{\pm 1}\Vert f_A\Vert _\infty \\&\le 3^d\text {BH}^{\le d}_{\pm 1}\Vert A\Vert , \end{aligned}$$

where in the first and last inequalities we used Proposition 2.2, and in the second inequality we used the Bohnenblust–Hille inequality on $\{-1,1\}^n$. $\square$

Note that Corollary 1.3 follows immediately from Theorem 1.2. Now we prove Corollary 1.6.

### Proof of Corollary 1.6

Fix $\eta >0$. Consider the following set

$$\begin{aligned} \mathcal {A}_\eta \,{:}{=}\,\left\{ {\textbf {s}}\in \{0,1,2,3\}^n:|\widehat{A}_{\textbf {s}}|>\eta \right\} . \end{aligned}$$

Recall that $\Vert A\Vert \le 1$. Then by Markov’s inequality and noncommutative Bohenblust–Hille inequality (denoting $|\mathcal {A}_\eta |$ the cardinality of $\mathcal {A}_\eta$)

$$\begin{aligned} |\mathcal {A}_\eta | \le \eta ^{-\frac{2d}{d+1}}\sum _{{\textbf {s}}\in \mathcal {A}_\eta }|\widehat{A}_{{\textbf {s}}}|^{\frac{2d}{d+1}} \le \eta ^{-\frac{2d}{d+1}}\left( \text {BH}^{\le d}_{M_2({\mathbb {C}})}\right) ^{\frac{2d}{d+1}}. \end{aligned}$$

Then $B_\eta \,{:}{=}\,\sum _{{\textbf {s}}\in \mathcal {A}_\eta }\widehat{A}_{{\textbf {s}}}\sigma _{{\textbf {s}}}$ depends on at most *k* coordinates with

$$\begin{aligned} k\le d|\mathcal {A}_\eta | \le d\eta ^{-\frac{2d}{d+1}}\left( \text {BH}^{\le d}_{M_2({\mathbb {C}})}\right) ^{\frac{2d}{d+1}}. \end{aligned}$$

Again, by noncommutative Bohenblust–Hille inequality and the fact that $\Vert A\Vert \le 1$

$$\begin{aligned} \Vert A-B_\eta \Vert _2^2 =\sum _{{\textbf {s}}\notin \mathcal {A}_\eta }|\widehat{A}_{\textbf {s}}|^2 \le \eta ^{\frac{2}{d+1}}\sum _{{\textbf {s}}\notin \mathcal {A}_\eta }|\widehat{A}_{\textbf {s}}|^{\frac{2d}{d+1}} \le \eta ^{\frac{2}{d+1}}\left( \text {BH}^{\le d}_{M_2({\mathbb {C}})}\right) ^{\frac{2d}{d+1}}. \end{aligned}$$

Now choose $B=B_\eta$ with

$$\begin{aligned} \eta \,{:}{=}\,\epsilon ^{d+1}\left( \text {BH}^{\le d}_{M_2({\mathbb {C}})}\right) ^{-d}. \end{aligned}$$

Then $\Vert A-B\Vert _2\le \epsilon$, where *B* is a *k*-junta with

$$\begin{aligned} k\le d\eta ^{-\frac{2d}{d+1}}\left( \text {BH}^{\le d}_{M_2({\mathbb {C}})}\right) ^{\frac{2d}{d+1}} =\epsilon ^{-2d}d\left( \text {BH}^{\le d}_{M_2({\mathbb {C}})}\right) ^{2d}. \end{aligned}$$

This finishes the proof, since $\text {BH}^{\le d}_{M_2({\mathbb {C}})}\le C^d$ for some universal $C>0$ by Theorem 1.2. $\square$

## Learning quantum observables of low degree

Let us review the classical learning model first. Suppose that we want to learn a class $\mathcal {F}$ of functions on $\{-1,1\}^n$ using the random query model which we shall explain. For $N\ge 1$, let $X_1,\ldots , X_N$ be *N* i.i.d. random variables uniformly distributed on $\{-1,1\}^n$. Then how many random queries do we need to recover functions $f\in \mathcal {F}$ nicely? More precisely, fix the error parameters $\epsilon ,\delta \in (0,1)$, what is the least number $N=N(\mathcal {F},\epsilon ,\delta )>0$ such that for any $f\in \mathcal {F}$ together with *N* random queries

$$\begin{aligned} (X_1,f(X_1)),\ldots , (X_N, f(X_N)) \end{aligned}$$

one can construct a random function $h:\{-1,1\}^n\rightarrow \mathbb {R}$ such that

$$\begin{aligned} \Vert f-h\Vert _2^2\le \epsilon \end{aligned}$$

with probability at least $1-\delta$ ? Here $\Vert f\Vert _2$ denotes the $L^2$-norm of *f* with respect to the uniform probability measure on $\{-1,1\}^n$. When $\mathcal {F}=\mathcal {F}^{\le d}_n$ consists of functions $f:\{-1,1\}^n\rightarrow [-1,1]$ of degree at most *d*, Eskenazis and Ivanisvili [14] proved that

$$\begin{aligned} N(\mathcal {F}^{\le d}_n,\epsilon ,\delta )\le \frac{1}{\varepsilon ^{d+1}} \Big (\log \frac{n}{\delta }\Big ) C(d), \end{aligned}$$

where *C*(*d*) depends on the Bohnenblust–Hille constant $\text {BH}^{\le d}_{\{\pm 1\}}$ for functions on $\{-1,1\}^n$ of degree at most *d*. So logarithmic number $\mathcal {O}_{\epsilon ,\delta ,d}(\log (n))$ of random queries is sufficient to learn bounded low-degree polynomials in the above sense. This improves significantly the previous work [17, 18] on the dimension-dependence of $N(\mathcal {F}^{\le d}_n,\epsilon ,\delta )$. Later on, Eskenazis et al. [15] proved that $\mathcal {O}_{\epsilon ,\delta ,d}(\log (n))$ is also necessary.

Now suppose that we want to learn quantum observable *A* over *n*-qubits, i.e. $A\in M_2({\mathbb {C}})^{\otimes n}$, of degree at most *d* with $\Vert A\Vert \le 1$.

Our learning model is similar to the classical setting. The random queries are now replaced with

$$\begin{aligned} \text {tr}[A\rho ],\qquad \rho \sim \mathcal {S}, \end{aligned}$$

where $\rho$ samples uniformly in some set $\mathcal {S}$ of density matrices in $M_2({\mathbb {C}})^{\otimes n}$. Our hope is to build another (random) observable ${\widetilde{A}}$ out of *N* random queries such that

4.1 $$\begin{aligned} \Vert {\widetilde{A}} -A\Vert _2^2\le \varepsilon \end{aligned}$$

with probability at least $1-\delta$. Again, the question is, how many random queries $N=N(\varepsilon , \delta , d, n)$ do we need to accomplish this, and how does *N* depend on *n*?

In the remaining part of this section we provide one answer to this question with $\mathcal {S}=\mathcal {S}_n$ constructed in Proposition 2.2.

### Theorem 4.1

Suppose that $A\in M_2({\mathbb {C}})^{\otimes n}$ is of degree at most *d* and $\Vert A\Vert \le 1$. Fix $\delta ,\epsilon \in (0,1)$ and

$$\begin{aligned} N\ge \frac{C^{d\sqrt{d\log d}}}{\epsilon ^{d+1}}\log \left( \frac{n}{\delta }\right) , \end{aligned}$$

with $C>0$ large enough. Then given any *N* random density matrices $\rho ^{(m)},1\le m\le N$ independently and uniformly sampled in $\mathcal {S}_n$, as well as random queries

$$\begin{aligned} \left( \rho ^{(m)}, \text {tr}[A\rho ^{(m)}]\right) ,\qquad \rho ^{(m)}\sim \mathcal {S}_n \end{aligned}$$

we can construct a random $\widetilde{A}\in M_2({\mathbb {C}})^{\otimes n}$ such that $\Vert A-\widetilde{A}\Vert _2^2\le \epsilon$ with probability at least $1-\delta$.

### Proof

Again, we will reduce the problem to commutative case, which we shall explain how. To any

$$\begin{aligned} A=\sum _{S\in \text {Im}(q)\subset 2^{[3n]}:|S|\le d} \widehat{A}_{p(S)}\sigma _{p(S)}\in M_2({\mathbb {C}})^{\otimes n}, \end{aligned}$$

we associate it with the function $f_A:\{-1,1\}^{3n}\rightarrow {\mathbb {C}}$ constructed in Proposition 2.2. Recall that $f_A$ is of degree at most *d*, $\Vert f_A\Vert _\infty \le 1$, and

$$\begin{aligned} f_A=\text {tr}[A\rho (\cdot )]=\sum _{S\in \text {Im}(q)\subset 2^{[3n]}:|S|\le d}3^{-|S|} \widehat{A}_{p(S)}\chi _S. \end{aligned}$$

Suppose that we have $\rho ({x}(m))\in \mathcal {S}_n,1\le m\le N$ as our independent random density matrices and

$$\begin{aligned} \left( \rho ({x}(m)), \text {tr}[A\rho ({x}(m))]\right) ,\qquad 1\le m\le N \end{aligned}$$

as random queries, where ${x}(m),1\le m\le N$ are i.i.d. random variables uniformly distributed on $\{-1,1\}^{3n}$. Similar to the commutative setting [15], we approximate

$$\begin{aligned} {\widehat{f}}_A(S)=3^{-|S|} \widehat{A}_{p(S)},\qquad S\in \text {Im}(q),\quad |S|\le d \end{aligned}$$

with the empirical Walsh coefficients

$$\begin{aligned} \alpha _S\,{:}{=}\, \frac{1}{N} \sum _{m=1}^N f_A(x(m)) \chi _S(x(m)) =\frac{1}{N} \sum _{m=1}^N\text {tr}[A\rho (m)] \chi _S(x(m)). \end{aligned}$$

Of course $\textbf{E}\alpha _S = {\widehat{f}}_A(S)$ where $\textbf{E}$ is with respect to the uniform distribution. Since $\alpha _S$ is the sum of i.i.d. random variables, we get by $\Vert f_A\Vert _\infty \le 1$ and the Chernoff bound that for $b>0$

4.2 $$\begin{aligned} \textbf{P}\left\{ |\alpha _S- {\widehat{f}}_A(S)| > b\right\} \le 2\exp \left( -N b^2/2\right) , \forall S\in \text {Im}(q), |S|\le d\,. \end{aligned}$$

Note that

$$\begin{aligned} |\{S\in \text {Im}(q):|S|\le d\}|&=|\{{\textbf {s}}\in \{0,1,2,3\}^n:|{\textbf {s}}|\le d\}|\\&=\sum _{l=0}^{d}3^l\left( {\begin{array}{c}n\\ l\end{array}}\right) \le 3^d\sum _{l=0}^{d}\left( {\begin{array}{c}n\\ l\end{array}}\right) . \end{aligned}$$

Then by (4.2) and the union bound

$$\begin{aligned}&\textbf{P}\left\{ \exists S\in \text {Im}(q), |S|\le d: |\alpha _S-{\widehat{f}}_A(S)|> b \right\} \\&\quad \le 2e^{-Nb^2/2} |\{S\in \text {Im}(q):|S|\le d\}|\\&\quad \le 2e^{-Nb^2/2}3^d \sum _{l=0}^{d}{n \atopwithdelims ()l}. \end{aligned}$$

Choosing

4.3 $$\begin{aligned} N\ge \frac{2}{b^2}\log \left( \frac{2\cdot 3^d}{\delta }\sum _{l=0}^{d}\left( {\begin{array}{c}n\\ l\end{array}}\right) \right) , \end{aligned}$$

one achieves

4.4 $$\begin{aligned} \textbf{P}\left\{ |\alpha _S- {\widehat{f}}_A(S)| \le b,\forall S\in \text {Im}(q), |S|\le d \right\} \ge 1-\delta . \end{aligned}$$

We continue to copycat [14] and introduce the random sets

$$\begin{aligned} \mathscr {S}_b\,{:}{=}\,\left\{ S\in \text {Im}(q),|S|\le d: |\alpha _S|\ge 2b\right\} . \end{aligned}$$

In view of (4.4), with probability $\ge 1-\delta$:

4.5 $$\begin{aligned} {\left\{ \begin{array}{ll} |\widehat{f}_A(S)|\le |\alpha _S|+|\alpha _S-\widehat{f}_A(S)|< 3b, &{} \text { if } S\notin {\mathscr {S}}_b\\ |\widehat{f}_A(S)|\ge |\alpha _S|-|\alpha _S-\widehat{f}_A(S)|\ge b, &{} \text { if } S\in {\mathscr {S}}_b \end{array}\right. }. \end{aligned}$$

The second line of (4.5), together with $\Vert f_A\Vert _\infty \le 1$ and the commutative Bohnenblust–Hille inequality (1.3), yields

$$\begin{aligned} |\mathscr {S}_b|\le b^{-\frac{2d}{d+1}}\sum _{S\in \mathscr {S}_b}|\widehat{f}_A(S)|^{\frac{2d}{d+1}} \le b^{-\frac{2d}{d+1}}\left( \text {BH}^{\le d}_{\{\pm 1\}}\right) ^{\frac{2d}{d+1}}. \end{aligned}$$

Fix *b* as above. Consider the random polynomial in $M_2({\mathbb {C}})^{\otimes n}$

$$\begin{aligned} A_{b}\,{:}{=}\,\sum _{S\in \mathscr {S}_b}3^{|S|}\alpha _S\sigma _{p(S)} =\sum _{q({\textbf {s}})\in \mathscr {S}_b}3^{|{\textbf {s}}|}\alpha _{q({\textbf {s}})}\sigma _{{\textbf {s}}}. \end{aligned}$$

All combined, we have with probability at least $1-\delta$ that

$$\begin{aligned} \Vert A-A_{b}\Vert _2^2&\le 3^{2d}\sum _{S\in \mathscr {S}_b}|\alpha _S-\widehat{f}_A(S)|^2 +3^{2d}\sum _{S\notin \mathscr {S}_b}|\widehat{f}_A(S)|^2\\&\le 3^{2d} b^2|\mathscr {S}_b|+3^{2d}(3b)^{\frac{2}{d+1}}\sum _{S\notin \mathscr {S}_b}|\widehat{f}_A(S)|^{\frac{2d}{d+1}}\\&\le \left( 3^{d+1}\text {BH}^{\le d}_{\{\pm 1\}}\right) ^{\frac{2d}{d+1}}\left( b^2\cdot b^{-\frac{2d}{d+1}}+(3b)^{\frac{2}{d+1}}\right) \\&\le 10\left( 3^{d+1}\text {BH}^{\le d}_{\{\pm 1\}}\right) ^{\frac{2d}{d+1}}b^{\frac{2}{d+1}}. \end{aligned}$$

To get an error bound $\Vert A-A_{b}\Vert _2^2\le \epsilon$, we choose

$$\begin{aligned} b=10^{-\frac{d+1}{2}}\left( 3^{d+1}\text {BH}^{\le d}_{\{\pm 1\}}\right) ^{-d}\epsilon ^{\frac{d+1}{2}}. \end{aligned}$$

Inserting this into (4.3), we choose *N* such that

$$\begin{aligned} N\ge \frac{2\cdot 10^{d+1}\left( 3^{d+1}\text {BH}^{\le d}_{\{\pm 1\}}\right) ^{2d}}{\epsilon ^{d+1}}\log \left( \frac{2\cdot 3^d}{\delta }\sum _{l=0}^{d}\left( {\begin{array}{c}n\\ l\end{array}}\right) \right) . \end{aligned}$$

Noting moreover that (see for example [14])

$$\begin{aligned} \sum _{l=0}^{d}\left( {\begin{array}{c}n\\ l\end{array}}\right) \le \left( \frac{en}{d}\right) ^d, \end{aligned}$$

we may choose

$$\begin{aligned} N\ge \frac{C^{d\sqrt{d\log d}}}{\epsilon ^{d+1}}\log \left( \frac{n}{\delta }\right) , \end{aligned}$$

for some $C>0$ large enough. Here, the Bohenblust–Hille constant $\text {BH}^{\le d}_{\{\pm 1\}}$ is contained in $C^{d\sqrt{d\log d}}$. With this choice of *N*, the random polynomial $\widetilde{A}\,{:}{=}\,A_{b}$ above satisfies

$$\begin{aligned} \Vert A-\widetilde{A}\Vert _2^2\le \epsilon , \end{aligned}$$

with probability $$\ge 1-\delta $$. $$\square $$

## Bohr’s radius phenomenon on quantum Boolean cubes

One important application of classical Bohnenblust–Hille inequalities is to study *Bohr’s radius problem* [5]. The original problem [2] concerns the *n*-dimensional torus $\mathbb {T}^n$ with $\mathbb {T}=\{z\in {\mathbb {C}}:|z|=1\}$ and the exact asymptotic behaviour of Bohr’s radius was obtained by Bayart et al. [7] using the polynomial version of Bohnenblust–Hille inequalities (1.2) with the best constants (denoted by $\text {BH}^{\le d}_{\mathbb {T}}$) of subexponential growth in the degree *d*. See also [9]. A Boolean analogue of the problem was studied by Defant et al. in [11], where Bohr’s radius is replaced by *Boolean’s radius*.

### Definition 5.1

*Boolean’s radius* of a function $f:\{-1,1\}^n\rightarrow \mathbb {R}$ is the positive real number $\text {Br}_n(f)$ such that

$$\begin{aligned} \sum _{S\subset [n]}|\widehat{f}(S)|\text {Br}_n(f)^{|S|}=\Vert f\Vert _\infty . \end{aligned}$$

Given a class $\mathcal {F}_n$ of functions on $\{-1,1\}^n$, the Boolean radius of $\mathcal {F}_n$ is defined as

$$\begin{aligned} \text {Br}_n(\mathcal {F}_n)\,{:}{=}\,\inf \{\text {Br}_n(f):f\in \mathcal {F}_n\}. \end{aligned}$$

Of particular interests to us are the following four classes of functions

1. $\mathcal {F}_n(\text {all})$: all real functions on $\{-1,1\}^n$ with
  $$\begin{aligned} \text {Br}_n(\text {all})\,{:}{=}\,\text {Br}_n(\mathcal {F}_n(\text {all})); \end{aligned}$$
2. $\mathcal {F}_n(\hom )$: all real homogeneous functions on $\{-1,1\}^n$ with
  $$\begin{aligned} \text {Br}_n(\hom )\,{:}{=}\,\text {Br}_n(\mathcal {F}_n(\hom )); \end{aligned}$$
3. $\mathcal {F}_n(=d)$: all *d*-homogeneous real functions on $\{-1,1\}^n$ with
  $$\begin{aligned} \text {Br}_n(=d)\,{:}{=}\,\text {Br}_n(\mathcal {F}_n(=d)); \end{aligned}$$
4. $\mathcal {F}_n(\le d)$: all real functions on $\{-1,1\}^n$ of degree at most *d* with
  $$\begin{aligned} \text {Br}_n(\le d)\,{:}{=}\,\text {Br}_n(\mathcal {F}_n(\le d)). \end{aligned}$$

The problem of Boolean’s radius is to determine the right order of decay of $\text {Br}_n(\mathcal {F}_n)$ as $n\rightarrow \infty$. Among others, Defant, Mastyło and Pérez proved the following.

### Theorem 5.2

[11, Theorems 2.1 & 3.1 & 4.1 and Corollary 3.2] For any $1\le d\le n$, we have

1. $$\text {Br}_n(\text {all})=2^{1/n}-1$$ and thus $$\lim \nolimits _{n\rightarrow \infty }n\text {Br}_n(\text {all})=\log 2$$;
2. there exists $C>1$ such that for all
  $$\begin{aligned} c_d n^{\frac{1}{2n}}\left( {\begin{array}{c}n\\ d\end{array}}\right) ^{-\frac{1}{2d}} \le \text {Br}_n(=d) \le C_d n^{\frac{1}{2n}}\left( {\begin{array}{c}n\\ d\end{array}}\right) ^{-\frac{1}{2d}}, \end{aligned}$$
  where
  $$\begin{aligned} c_d=\frac{1}{d^{\frac{1}{2d}}C^{\sqrt{\frac{\log d}{d}}}} \qquad \textrm{and } \qquad C_d=C^{\frac{1}{d}}; \end{aligned}$$
3. $$\lim \nolimits _{n\rightarrow \infty }\sqrt{\frac{n}{\log n}}\text {Br}_n(\hom )=1$$;
4. there exist $c'_d, C'_d>0$ such that
  $$\begin{aligned} \frac{c'_d}{n^{\frac{1}{2}}} \le \text {Br}_n(\le d) \le \frac{C'_d}{n^{\frac{d-1}{2d}}}. \end{aligned}$$

We refer to [11] for more discussions on the similarity and differences between the Boolean cube case and torus case.

The concept of Boolean radius carries over to the quantum setting without any difficulties.

### Definition 5.3

The *quantum Boolean radius* of self-adjoint $A\in M_2({\mathbb {C}})^{\otimes n }$ is the positive real number $\text {qBr}_n(A)$ such that

$$\begin{aligned} \sum _{{\textbf {s}}\in \{0,1,2,3\}^n}|\widehat{A}_{\textbf {s}}|\text {qBr}_n(A)^{|{\textbf {s}}|}=\Vert A\Vert . \end{aligned}$$

Similarly, one may define for a class $\mathcal {F}_n'$ of self-adjoint matrices in $M_2({\mathbb {C}})^{\otimes n }$

$$\begin{aligned} \text {qBr}_n(\mathcal {F}_n')\,{:}{=}\,\inf \{\text {qBr}_n(A):A\in \mathcal {F}_n'\}. \end{aligned}$$

### Remark 5.4

In [11] only the real-valued functions were considered and some arguments rely on the real structure. We are not going to discuss the possible extension to the complex-valued functions here. It is for this reason we require *A* to be self-adjoint. In fact, when *A* is self-adjoint, the function $f_A=\text {tr}[A\rho (\cdot )]$ on $\{-1,1\}^{3n}$ constructed in Proposition 2.2 is real-valued and has real Fourier coefficients, so that we can use the results in [11] directly. We leave the problem for general *A* to future study.

If $\mathcal {F}_n$ denotes one of the four classes of functions (1–4) on $\{-1,1\}^n$ listed as above, then we use $\mathcal {F}_n^q$ to denote the quantum counterpart of class of matrices in $M_2({\mathbb {C}})^{\otimes n}$. For example, if $\mathcal {F}_n=\mathcal {F}_n(\le d)$ is the class of polynomials on $\{-1,1\}^n$ of degree at most *d*, then $\mathcal {F}_n^q=\mathcal {F}^q_n(\le d)$ denotes the class of self-adjoint $A\in M_2({\mathbb {C}})^{\otimes n}$ of degree at most *d*. Then our main result on quantum Boolean radius is the following.

### Theorem 5.5

For any $1\le d\le n$ and any $\mathcal {F}_n$ of the four classes of functions (1–4) listed above, we have

$$\begin{aligned} \text {qBr}_n(\mathcal {F}_n^q)\le \text {Br}_n(\mathcal {F}_n)\qquad \textrm{and }\qquad \text {Br}_{3n}(\mathcal {F}_{3n})\le 3\text {qBr}_{n}(\mathcal {F}_{n}^q). \end{aligned}$$

### Proof

The first inequality is trivial, as $\mathcal {F}_n$ can be viewed as a subset of $\mathcal {F}^q_n$ with all the relevant structures (norm, degree etc.) preserved. In fact, for any $f:\{-1,1\}^n\rightarrow \mathbb {R}$ with Fourier expansion

$$\begin{aligned} f(x_1,\ldots ,x_n)=\sum _{0\le l\le n}\sum _{1\le i_1<\cdots <i_l\le n} a_{i_1,\ldots , i_l}x_{i_1}\ldots x_{i_l}, \end{aligned}$$

consider the self-adjoint matrix

$$\begin{aligned} A_f=\sum _{0\le l\le n}\sum _{1\le i_1<\cdots <i_l\le n} a_{i_1,\ldots , i_l}\sigma _{i_1,\ldots , i_l}^{3,\ldots , 3}. \end{aligned}$$

Clearly, $\deg (f)=\deg (A_f)$ and $A_f\in \mathcal {F}^q_n$ whenever $f\in \mathcal {F}_n$. Note that the canonical basis of $M_2({\mathbb {C}})$ are eigenvectors of $\sigma _3$ corresponding to eigenvalues $1,-1$, respectively. Let us denote this basis $\{e_1,e_{-1}\}$. Then under the basis $\{e_{x_1}\otimes \cdots \otimes e_{x_n}: x_j\in \{-1,1\},1\le j\le n\}$, $A_f$ is a diagonal matrix with diagonal entries being

$$\begin{aligned} \langle A_f(e_{x_1}\otimes \cdots \otimes e_{x_n}),e_{x_1}\otimes \cdots \otimes e_{x_n}\rangle =\sum _{0\le l\le n}\sum _{1\le i_1<\cdots <i_l\le n} a_{i_1,\ldots , i_l}x_{i_1} \ldots x_{i_l}. \end{aligned}$$

So $\Vert f\Vert _\infty =\Vert A_f\Vert$. By definition, we have $\text {Br}_n(f)=\text {qBr}_n(A_f)$ and this proves the first inequality.

To prove the second inequality, we appeal to our reduction method again. In fact, take any $A=A^*\in \mathcal {F}_n^q\subset M_2({\mathbb {C}})^{\otimes n}$. Consider the function $f_A:\{-1,1\}^{3n}\rightarrow \mathbb {R}$ constructed in Proposition 2.2 which belongs to $\mathcal {F}_{3n}$ whenever $\mathcal {F}_n$ is one of four aforementioned classes (1–4). Take the class (3) $\mathcal {F}_n(=d)$ for example. By construction, $f_A$ is *d*-homogeneous if *A* is. Recall that $\Vert f_A\Vert _\infty \le \Vert A\Vert$, and by definition of $\text {qBr}_n(A)$:

$$\begin{aligned} \sum _{{\textbf {s}}}|\widehat{A}_{\textbf {s}}|\text {qBr}_n(A)^{|{\textbf {s}}|} =\sum _{S\in \text {Im}(q)\subset 2^{[3n]}}|\widehat{A}_{p(S)}|\text {qBr}_n(A)^{|S|} =\Vert A\Vert . \end{aligned}$$

Then for $f_A:\{-1,1\}^{3n}\rightarrow \mathbb {R}$, we have

$$\begin{aligned} \sum _{S\in \text {Im}(q)\subset 2^{[3n]}}|\widehat{f}_A(S)|(3\text {qBr}_n(A))^{|S|}&=\sum _{S\in \text {Im}(q)\subset 2^{[3n]}}|\widehat{A}_{p(S)}|\text {qBr}_n(A)^{|S|}\\&=\Vert A\Vert \ge \Vert f_A\Vert _{\infty }. \end{aligned}$$

Therefore, by definition of $\text {Br}_{3n}(f_A)$:

$$\begin{aligned} \text {Br}_{3n}(f_A)\le 3\text {qBr}_n(A). \end{aligned}$$

So for any $A=A^*\in \mathcal {F}_n^q$ we find $f_A\in \mathcal {F}_{3n}$ such that the above inequality holds. Therefore we get

$$\begin{aligned} \text {Br}_{3n}(\mathcal {F}_{3n})\le 3\text {qBr}_n(\mathcal {F}_n^q) \end{aligned}$$

by definitions of $\text {Br}_{3n}(\mathcal {F}_{3n})$ and $\text {qBr}_n(\mathcal {F}_n^q)$. This concludes the proof of the second inequality. $\square$

### Remark 5.6

It is still possible to improve our estimates using the reduction method. For example, we have shown that for the class $\mathcal {F}_n^q(\text {all})$ of all self-adjoint matrices in $M_2({\mathbb {C}})^{\otimes n}$

$$\begin{aligned} \frac{2^{1/(3n)}-1}{3}\le \text {qBr}_n(\mathcal {F}_n^q(\text {all}))\le 2^{1/n}-1. \end{aligned}$$

But we can actually prove

$$\begin{aligned} \frac{2^{1/n}-1}{9}\le \text {qBr}_n(\mathcal {F}_n^q(\text {all}))\le 2^{1/n}-1. \end{aligned}$$

In fact, for any $A=A^*\in M_2({\mathbb {C}})^{\otimes n}$ with $\Vert A\Vert =1$, suppose $f_A:\{-1,1\}^{3n}\rightarrow [-1,1]$ is constructed as before. Then for any ${\textbf {0}}\ne {\textbf {s}}=p(S)\in \{0,1,2,3\}^n$, we have by [11, Lemma 2.2] that

$$\begin{aligned} |\widehat{A}_{\textbf{0}}|+ 3^{-|S|}|\widehat{A}_{p(S)}| =|\widehat{f}_A(\emptyset )|+ |\widehat{f}_A(S)| \le 1. \end{aligned}$$

So we get

$$\begin{aligned} \sum _{{\textbf {s}}\in \{0,1,2,3\}^n}|\widehat{A}_{{\textbf {s}}}|r^{|{\textbf {s}}|}&\le |\widehat{A}_{\textbf{0}}|+(1- |\widehat{A}_{\textbf{0}}|)\sum _{{\textbf {s}}\ne \textbf{0}}(3r)^{|{\textbf {s}}|}\\&\le |\widehat{A}_{\textbf{0}}|+(1- |\widehat{A}_{\textbf{0}}|)\sum _{k=1}^{n}\left( {\begin{array}{c}n\\ k\end{array}}\right) (9r)^{k}\\&= |\widehat{A}_{\textbf{0}}|+(1- |\widehat{A}_{\textbf{0}}|)\left( (1+9r)^n-1\right) . \end{aligned}$$

This establishes the bound $\text {qBr}_n(\mathcal {F}_n^q(\text {all}))\ge \frac{2^{1/n}-1}{9}$ by definition.

## Discussions

We briefly compare our results with the work of Huang et al. [16].

1. In the work [16], the noncommutative Bohnenblust–Hille inequality (1.4) [16, Corollary 3] follows from a more general result [16, Theorem 5] which we do not have. The noncommutative Bohnenblust–Hille constant ($C_d$ in (1.4)) we obtained is of exponential growth $\sim C^d$, which is better than theirs $\sim d^{\mathcal {O}(d)}$. Remark that the best known bound for the (commutative) Boolean cubes is subexponential $\sim C^{\sqrt{d\log d}}$ [12].
2. Our proof of (1.4) is different from theirs. We use an argument that reduces the problem to the commutative case and the proof looks simpler, while their proof that is more self-contained, combines several technical estimates that can be useful to other problems.
3. Our better Bohnenblust–Hille constant yields an immediate improvement for learning quantum observable up to a small prediction error in their work. In particular, this allows us to remove the $\log \log (1 / \epsilon )$ factor in the exponent of [16, Eq. (A17)]. It also yields an immediate improvement in the sample complexity for learning arbitrary quantum process. This is because the learning is achieved by considering the unknown observable to be the observable after Heisenberg evolution under the unknown quantum process.

## Data Availability

All data generated or analysed during this study are included in this article.
